# Normalized Interferon Signatures and Clinical Improvements by IFNAR1 Blocking Antibody (Anifrolumab) in Patients with Type I Interferonopathies

**DOI:** 10.1007/s10875-024-01826-2

**Published:** 2024-10-23

**Authors:** Genia Kretzschmar, Laura Piñero Páez, Ziyang Tan, Jun Wang, Laura Gonzalez, Constantin Habimana Mugabo, Anette Johnsson, Yang Chen, Jaromír Mikeš, Tadepally Lakshmikanth, Anna James, Raphaela Goldbach-Mansky, Marie Fischer, Karin Palmblad, Sara Alehashemi, AnnaCarin Horne, Petter Brodin

**Affiliations:** 1 Unit for Clinical Pediatrics, Dept. of Women’s and Children’s Health, Karolinska Institutet, 17165 Solna, Sweden; 2 Unit for Pediatric Rheumatology, Astrid Lindgren’s Children’s Hospital, Karolinska University Hospital, Solna, Sweden; 3 National Institute of Allergy and Infectious Diseases, NIH, Bethesda, MD 20892 USA; 4 Department of Medicine, Rheumatology Unit, Karolinska Institutet, Stockholm, Sweden; 5 Department of Immunology and Inflammation, Imperial College London, London, W12 0NN UK; 6 Medical Research Council, Laboratory of Medical Sciences (LMS), Imperial College Hammersmith Campus, London, UK

**Keywords:** IFN-I, type I IFN, IFNAR1, Interferonopathies, Anifrolumab

## Abstract

**Purpose:**

A causal role of type-I interferons (IFN-I) in autoinflammatory type-I interferonopathies such as SAVI (STING–associated vasculopathy with onset in infancy) and CANDLE (chronic atypical neutrophilic dermatosis with lipodystrophy and elevated temperatures) is suggested by elevated expression of IFN-I stimulated genes (ISGs). Hitherto, the lack of specific inhibitors of IFN-I signaling has prevented the verification of a causal role for IFN-I in these conditions. Commonly used inhibitors of the JAK/STAT pathway exert broad effects on multiple signaling pathways leading to more general immunosuppression beyond IFN-I signaling.

**Methods:**

Here we show in four patients with SAVI and one patient with CANDLE syndrome that blockade of the IFNAR1 receptor (Anifrolumab) exerts an additive effect over JAK-inhibitor alone. In two patients with SAVI, monotherapy with Anifrolumab is sufficient to retain a suppressed IFN-I signature and clinical improvement.

**Results:**

Anifrolumab normalizes IFN-I signature genes and relieves symptoms beyond what is typically achieved by a JAK-inhibitor (Baricitinib) alone in patients with type-I interferonopathies. In two patients Anifrolumab was used successfully as monotherapy. Addition of Anifrolumab enabled steroid tapering and cessation with reduced overall immunosuppression and lower risks of opportunistic infections and improved metabolic states and growth which is highly beneficial in these young patients.

**Conclusion:**

These results verify a causal role of IFN-I signaling in type-I Interferonopathies SAVI and CANDLE and suggests Anifrolumab as an important new treatment option in autoinflammatory diseases with elevated IFN-I induced gene expression.

Genia Kretzschmar, Laura Piñero Páez, and Ziyang Tan are shared-first authors.

Sara Alehashemi, AnnaCarin Horne, and Petter Brodin are co-senior author.

## Introduction

Type I interferonopathies is a group of autoinflammatory disorders which present with fever, sterile inflammation and variable organ involvement and upregulated IFN-I induced gene expression which distinguish these conditions from classical IL-1-mediated autoinflammatory diseases. These disorders are caused by mutations that perturb type-I IFN regulation and was therefore called type-I interferonopathies [1], but a causal role for IFN-I molecules in these diseases have been impossible to prove in the absence of selective inhibitors of IFN-I induced signalling. In 2003 Crow, et al. noted functional overlap between Mendelian encephalopathy patients with Aicardi-Goutiéres syndrome (AGS), systemic lupus erythematosus (SLE) and viral infections by showing highly upregulated IFNa protein levels [2]. Following this, SAVI [3] and CANDLE syndrome [4] were described and multiple underlying genetic causes for these have been described [5, 6]. The known mendelian causes of type-I interferonopathies can be grouped into gain-of-function (GOF) mutations in nucleic acid sensor pathways (ex. DDX58 and IFIH1) or adaptor molecules within nucleic acid sensor pathways (ex. *STING1*), loss of function (LOF) mutations in RNA-editing enzymes (ex *ADAR1),* nucleic acid metabolizing enzymes (ex. *RNASEH2A, RNASEH2B, RNASEH2C, TREX1, SAMHD1, DNASE2*) or LOF mutations in negative regulators of IFN response pathways (*ISG15* and *USP18*). Finally, GOF mutations in IFN responsive pathways such as *STAT1*, *STAT2* can present with similar phenotypes [7].

Functionally these diverse conditions lead to increased IFN-I production which is believed to underlie inflammatory symptoms with different tissue involvement in different conditions. The most common therapy for monogenic IFN–mediated autoinflammatory diseases is the small molecule JAK1/2 inhibitor Baricitinib [8]. This therapy can be highly effective but exerts broader effects beyond the IFN-I pathway and is associated with treatment-emergent infections in most patients [8]. Anifrolumab (Saphnelo®, Astra Zeneca AB), is a humanized IgG1k monoclonal antibody therapy that binds to a subunit of the IFN-I receptor (IFNAR1) and was recently approved to treat adult patients with Systemic Lupus Erythematosus (SLE) [9, 17, 18, 19, 20]. Doroudchi et al., recently described the use of Anifrolumab in a patient with DNASE2 deficiency and elevated IFN-I [10] and an ongoing study (NCT02974595) is assessing this treatment for diverse sets of IFN-I mediated autoinflammatory diseases [11]. Here we report our experiences of using Anifrolumab in four patients with SAVI and one patient with CANDLE syndrome.

## Results

**Fig. 1:**
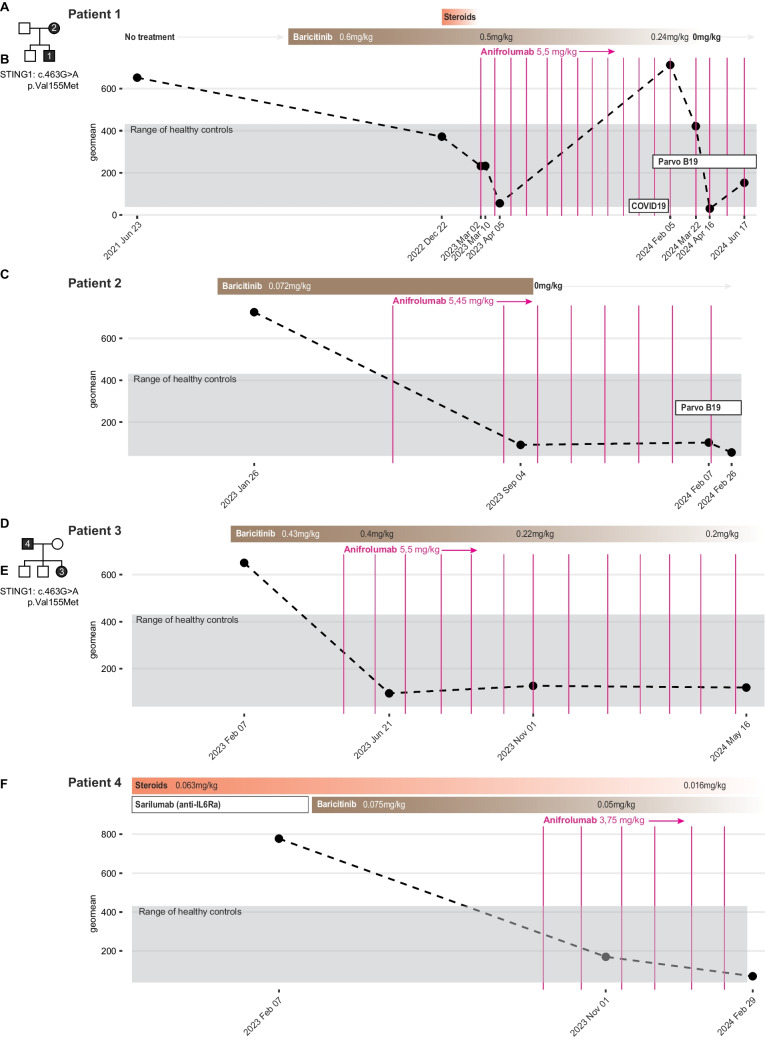
Immunomodulatory therapy in patients with type-I interferonopathy SAVI. (**A**) Pedigree of family 1 with STING-associated vasculopathy in infancy (SAVI) due to the indicated STING1 gain-of-function mutation. (**B**) Immunomodulatory therapy and (doses in mg/kg/day) and IFN-I-stimulated gene (ISG) expression as measured by Nanostring nCounter and presented as geometric mean (geomean) of normalized counts across n = 30 ISGs in whole blood stabilized in PaxGene at the time of blood collection. (**C**) Mother of index patient in family 1 also affected by SAVI and interstitial lung disease. (**D**) Pedigree of family 2 with SAVI due to the indicated STING1 gain-of-function mutation. (**E**) Immunomodulatory therapy and (doses in mg/kg/day) and IFN-I-stimulated gene (ISG) expression as measured by Nanostring nCounter and presented as geometric mean (geomean) of normalized counts across n = 30 ISGs in whole blood stabilized in PaxGene at the time of blood collection in affected daughter and, (**F**) father of the index case in family 2

A four-year-old boy was referred to us at the Pediatric rheumatology unit, Karolinska University hospital due to interstitial lung disease and a history of repeated chest infections requiring hospitalization and a status of low effort and activity level and frequent, prolonged cough episodes. The child was first of two to non-consanguineous parents of Swedish ancestry where the mother had a long history of chronic pulmonary disease of unknown cause with interstitial inflammation, cysts and fibrosis and under consideration for lung transplantation. Whole genome sequencing was performed in the index case, his brother and parents revealing an autosomal dominant, GOF variant in *STING1* (c.463G > A, p.Val155Met) verifying the diagnosis of SAVI in both index case and his mother (Fig. 1A). High-resolution computer tomography (HRCT) at diagnosis in the index case showed extended mediastinal lymphadenopathy and minor interstitial changes to the parenchyma but no air trapping. A 30-gene panel of IFN-I-induced genes was analysed prior to any treatment and revealed markedly elevated geometric mean expression in the index case (Fig. 1B). We were unable to secure a pre-treatment sample from the mother (Patient 2) but both patients were started on Baricitinib as previously described for patients with SAVI [8]. Follow-up molecular analyses of blood ISG six months later in the index case revealed markedly reduced levels of ISG from the time of diagnosis (Fig. 1B), but based on the experiences of the Goldbach-Mansky group at the National Institutes of Health (NIH) Anifrolumab (5.5 mg/kg) was added together with Baricitinib to both patients (Fig. 1B-C). Anifrolumab was well tolerated in monthly doses and a repeat ISG revealed further suppression over Baricitinib alone in both cases (Fig. 1B-C). In patient 1 the doses of Baricitinib were tapered from the initial 0.6 mg/kg/d to 0.24 mg/kg/d and steroids were rapidly discontinued. A repeat HRCT in patient 1 showed cysts as before, but reduced interstitial inflammatory lesions, and regression of the mediastinal lymphadenopathy when compared to a diagnostic test prior to either therapy. Similarly, patient 2 had a markedly reduced ISG following two doses of Anifrolumab and was able to taper steroids completely (Fig. 1C). Both patient 1 and 2 have since discontinued Baricitinib and are maintained on monotherapy Anifrolumab with continued silencing of ISGs although ISGs increased markedly following COVID-19 in patient 1 on continuous Anifrolumab therapy and both patients 1 and 2 have since had longstanding Parvovirus B19 infections (Fig. 1B-C). The overall clinical response in patient 1 has been remarkable with a 6-min walking test prior to treatment resulting in 330-m distance with oxygen desaturation to 77% while a repeat test following Baricitinib and Anifrolumab (May 2023) resulted in 420-m distance and no desaturation, and a recent repeat test on Anifrolumab in monotherapy (August 2024) resulted in 480 m of walking without desaturation which is within the normal range for healthy children of the same age (6 years).

A second family with a 5-year-old girl was referred to the Pediatric rheumatology unit, Karolinska University hospital, with suspected vasculitis and lung involvement and was treated with B-cell depleting therapy (anti-CD20, Rituximab), but subsequently was found to have a *STING1* (c.463G > A, p.Val155Met) mutation confirming the diagnosis of SAVI (Fig. 1D). This second family consists of two unaffected brothers, the index case and her non-consanguineous parents of Swedish ancestry with her 34-year-old father having a long history of undefined inflammatory disease involving joints, and lungs. The father carries the same autosomal dominant *STING1* variant causing SAVI and both patients 3 and 4 was started on Baricitinib and subsequently Anifrolumab (Fig. 1E-F). Molecular analyses of ISGs in family 2 index case revealed nearly complete silencing of ISGs following two doses of Anifrolumab together with Baricitinib (Fig. 1E) and also strong suppressive effects also in the father (Fig. 1F). Patient 3 has been able to taper Baricitinib doses markedly while retaining suppressed ISGs and patient 4 was able to taper both Baricitinib and steroid doses after Anifrolumab introduction. These observations suggest that Anifrolumab is a valuable treatment option in children and adults with SAVI leading to more efficient suppression of ISG as compared to Baricitinib alone and permits tapering of both Baricitinib and steroids with less general immunosuppression, better growth in young children and more precise targeting of IFN-I mediated signals as a result.

**Fig. 2:**
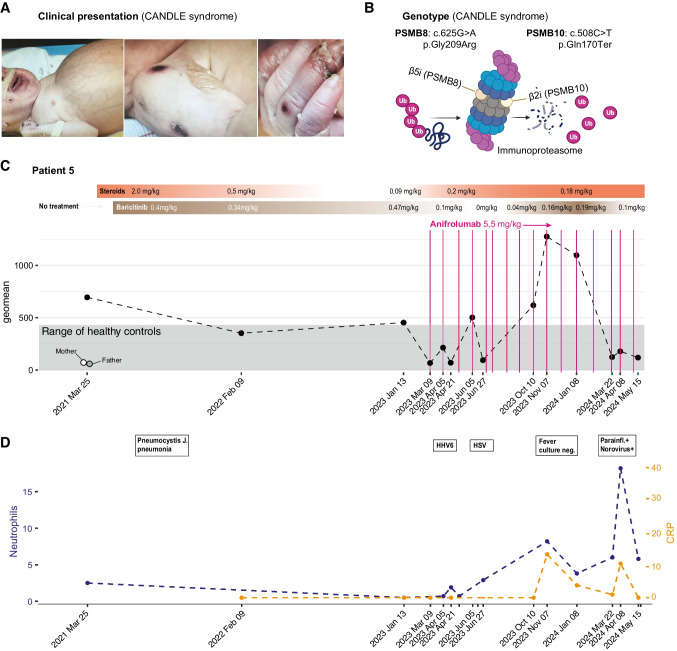
Immunomodulatory therapy in a child with CANDLE syndrome. (**A**) Photos taken shortly after birth showing distended abdomen and necrotic skin lesions in a newborn girl with hyperinflammation and multiorgan involvement. (**B**) digenic cause of disease with probable loss of function variants in two proteasomal genes.(**C**) Immunomodulatory therapy and IFN-I stimulated gene expression (ISG) in fourteen consecutive blood samples shown as geometric mean (geomean) of normalized counts across n = 30 ISGs shown over time. Samples from unaffected parents shown and shaded area represent ISG levels in healthy contol samples. The indicated immunomodulatory medication and doses in mg/kg/day. Vertical pink lines represent infusions of Anifrolumab. (**D**) Episodes of infection or possible infection/disease flare as indicated by Neutrophil count (10^9^/L of blood) and C-reactive protein (CRP)

Patient 5 is a complex case with severe, multiorgan involvement and type-I interferonopathy. This girl was born in Stockholm in gestational week 32 and small for gestational age with rapid development of fever and a hyperinflammatory syndrome with liver and kidney involvement, distended abdomen with ascites and necrotic skin lesions (Fig. 2A). During workup an elevated ISG was noted using the same 30-gene panel as described above [12] and WGS revealed two mutations within components of the immunoproteasome, *PSMB8 (c.625G* > *A, p.Gly209Arg)* and *PSMB10 (c.508C* > *T, p.Gln170Ter)*. The functional significance of these variants in this particular patient is unknown but other mutations in these same genes have previously been shown to underlie CANDLE syndrome [5, 13, 14] (Fig. 2B). This condition is caused by failure of the immunoproteasome to remove ubiquitinated proteins, leading to their intracellular accumulation and likely activation of the unfolded-protein response pathway and IFN-I induction (Fig. 2B) [15].

This critically ill newborn was treated with high-dose steroids and high-dose Baricitinib (0.4 mg/kg/d) as described for CANDLE syndrome [8]. Steroids alone efficiently clearing the skin lesions while steroids together with Baricitinib reduced the molecular ISG signature as compared to the pre-treatment baseline ISG. Dosing of Baricitinib in young children is difficult due to very efficient liver metabolism and persistent steroid use prevented stature growth in this child, while inadequate control of the underlying inflammation likely attribute to the developmental delays seen during the first year of life (Fig. 2C). The severe immunosuppression of combined Baricitinib and high-dose steroids also led to severe opportunistic infections such as a pneumocystis pneumonia. At two years of age Anifrolumab was added to further suppress IFN-I induced genes and better control the inflammation and hopefully allow tapering of Baricitinib. ISGs were much reduced in three follow-up measurements on Baricitinib and steroids in combination with Anifrolumab, as compared to Baricitinib and steroids alone (Fig. 2C). Following this initial molecular response, after tapering of Baricitinib, the ISG rose again in June 2023 following primary infections with Human Herpes Virus 6 (HHV6) and Herpes simplex virus (HSV) infections (Fig. 2C). After discontinuing Baricitinib and steroids there was massive upregulation of ISGs and also an episode of fever without detection of any infectious agents in November/December 2023 leading to dose escalation of steroids and Baricitinib (Fig. 2C-D). This child with CANDLE has required the combined triple therapy (Anifrolumab, Baricitinib and Steroids) since in order to prevent disease flares although doses of steroids and Baricitinib have been kept at a minimum to avoid growth restriction and overall immunosuppression and opportunistic infections.

**Fig. 3:**
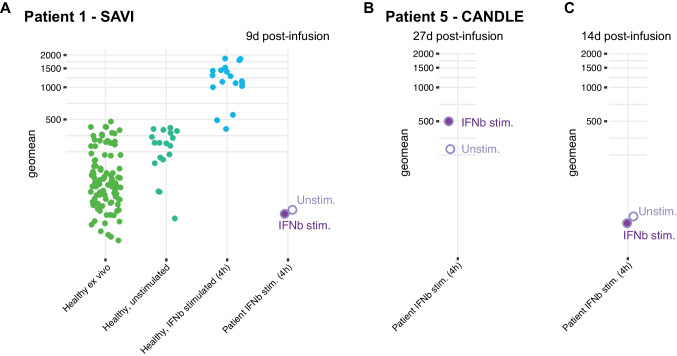
Responsiveness to IFN-I in vitro during Anifrolumab treatment. (**A**) ISG measure by Nanostring nCounter using untreated blood from healthy controls (green), Unstimulated healthy donor blood cultured in vitro, IFNb stimulated blood from healthy controls, and blood from Patient 1 (SAVI) collected during daily Baricitinib (0.5 mg/kg/d) and 9 days following Anifrolumab infusion (5.5 mg/kg). The sample was either IFNb stimulated or unstimulated prior to ISG measurement revealing no upregulation of ISG following IFNb stimulation in patient blood. (**B**) Patient 5 (CANDLE) during Baricitinib (0.1 mg/k- g/d), steroids (0.2 mg/kg/d of prednisolone eqvivalent) and 5.5 mg/kg Anifrolumab therapy on April 5th, 2023 (27 days post infusion) or (**C**) April 21st, 2023 (14 days post infusion)

To further assess the suppression of IFN-I induced gene expression we compared the 30-gene ISG in healthy controls ex vivo, to healthy control blood samples stimulated for 4 h in vitro with IFNb showing the expected upregulation of ISG (Fig. 3A). We then assessed the ISG response to IFNb stimulation in blood collected from patient 1 (SAVI) during combined Baricitinib (0.5 mg/kg/d) and Anifrolumab (5.5 mg/kg monthly) therapy. The sample was collected on April 5th, 2023, 9 days after last Anifrolumab infusion and we found no induction of ISGs following IFNb stimulation (Fig. 3A). In Patient 5 (CANDLE) with more intense immunomodulatory therapy and a more fluctuating disease course we tested two consecutive samples during the same triple therapy regimen with steroids (0.2 mg/kg/d), Baricitinib (0.1 mg/kg/d) and Anifrolumab (5.5 mg/kg monthly). In a sample collected 27 days following last Anifrolumab infusion the ISG was higher without IFNb stimulation (ISG: 282) as compared to a sample collected 14 days post infusion (ISG: 77) and following IFNb stimulation the score increased to 499 while the IFNb stimulation of blood collected 14 days post Anifrolumab showed no induction (ISG: 61)(Fig. 3B-C). These results indicate that in vitro stimulation of blood with IFN-I can further reveal the degree of IFN-I suppression by a given regimen in patients with type-I Interferonopathies.

Here we have described four patients with SAVI and one patient with CANDLE syndrome treated with Anifrolumab in combination with standard Baricitinib and steroids to more efficiently and selectively inhibit IFN-I mediated signals believed to underlie disease. Two SAVI patients were able to discontinue steroids and Baricitinib completely, all patients were able to reduce doses of Baricitinib and steroids as a consequence of adding Anifrolumab. Overall clinical responses were favourable with little adverse events associated with Anifrolumab and reducing doses of Baricitinib and steroids was associated with less opportunistic infections and better growth. To our knowledge this is the first description of this therapy as an addition to Baricitinib and Steroids in patients with type-I Interferonopathies although monotherapy with Anifrolumab was recently described in a patient with DNASE2 deficiency[10]. A larger study is ongoing (NCT02974595) that will determine best practices for use of Anifrolumab in different forms of type-I interferonopathies with longer follow-up time [11].

## Materials and Methods

### Patients

All patients were referred to and cared for at the Astrid Lindgren’s Children’s Hospital and adult Rheumatology centre at Karolinska University Hospital, Stockholm Sweden. Blood sampling and clinical data collection was approved by Swedish ethical review authority (ID: 2023–06579-01 and 2017/1964–31).

### Sample Processing

Blood drawn in an EDTA tube is, as soon as possible, aliquoted and mixed with PAXgene buffer in a 100:276 blood to PAXgene ratio, i.e. for 1 ml of blood, 2.76 ml of PAXgene buffer are used. After thoroughly mixing, the PAXgene sample is left at RT for a minimum of 1 h to ensure blood cell lysis and then frozen at -80ºC until its use for RNA isolation.

### RNA Isolation

The PAXgene sample previously frozen at -80ºC is thawed and equilibrated at RT for 30 min—1 h. Then, the protocol from PreAnalytix for the automated RNA purification from PAXgene samples is followed. Briefly, the sample is centrifuged twice, and the cell pellet resuspended in a buffer provided in the PAXgene kit. The following column-based purification steps for RNA isolation are performed automatically in the QIAcube (liquid handling platform from QIAGEN). Two aliquots of the eluted RNA are taken for the subsequent concentration measurement and integrity check. The remaining sample is frozen at -80ºC until its use for gene expression analyses.

### Gene Expression Analyses

The gene expression levels of 56 immune-related genes and 3 housekeeping genes are measured using NanoString Technologies. For this, a hybridization reaction between the mRNA molecules in the sample and a set of oligonucleotide probes, designed to capture the specific genes of interest, is carried out following NanoString’s recommendations. Briefly, around 5 µl of RNA sample are mixed with the oligonucleotide probes and incubated in a thermocycler at 65ºC, with a heated lid at 70ºC, for 20 h. Once the reaction time is completed, the sample is loaded into a cartridge designed to be read by NanoString’s nCounter instrument. The cartridge is then placed inside the nCounter and the gene expression assay is carried out within the instrument, which in the end provides a readout with raw mRNA counts of the genes of study.

Clinical samples, along with healthy donor reference samples, are run in the nCounter in batches of 12.

### Gene Expression Data Analysis

First, a quality check of the data is done by the nSolver software provided by NanoString. Then, as recommended by Nanostring, the data is pre-processed in two different normalization steps:

1. Internal positive control normalization.
2. Housekeeping genes normalization.

After normalization, two different scores (Z-score and geomean score) are calculated to provide a summary of the expression levels of type I IFN–stimulated genes (ISG scores), NF-κB–regulated genes (NF-κB scores) and type II IFN–regulated genes (IFN-γ scores) [12, 16].

Of note, NanoString’s products, along with any assays developed with its components are intended for research purposes only.

The normalization of nanostring gene counts follows the gene expression data analysis guidelines provided by NanoString Technologies. In brief, the raw counts from each sample were normalized by two normalization factors, the positive control factor and the housekeeping gene factor using the following formula,

$${factor}_{i}= \frac{\frac{1}{n}{\sum }_{i=1}^{n}{geomean}_{i}}{{geomean}_{i}}$$

In the formula, $i$ is the $i$-th sample, $n$ is the total number of samples and ${geomean}_{i}$ is the geometric mean of 6 positive controls in the $i$-th sample for the positive control factor, or 3 housekeeping genes (*NRDC*, *OTUD5*, *TUBB*) for the housekeeping gene factor. The normalized gene counts for each sample were calculated by multiplying the raw counts with the two factors.

## Data Availability

All scripts and data to reproduce the figures in the manuscript are available through our dedicated GitHub repository: https://github.com/Brodinlab/said_nanostring. The ethical permit does not permit us to share raw WGS data via public repositories, but we will also share such data upon establishment of a data transfer agreement. For inquiries contact Prof. P Brodin (petter.brodin@ki.se).
